# Phosphate scarcity governs methane production in the global open ocean

**DOI:** 10.1073/pnas.2521235123

**Published:** 2026-03-17

**Authors:** Shengyu Wang, Hairong Xu, Thomas S. Weber

**Affiliations:** ^a^Department of Earth and Environmental Sciences, University of Rochester, Rochester, NY 14627

**Keywords:** methane cycle, Marine Methane Paradox, aerobic methanogenesis, climate feedback

## Abstract

The observed supersaturation of methane (CH_4_) in open-ocean surface waters implies widespread CH_4_ production within the well-oxygenated mixed layer, driving emissions of this potent greenhouse gas to the atmosphere. The dominant CH_4_ production pathway that explains this phenomenon remains poorly understood, although candidates include production during photosynthesis, zooplankton metabolism, and dissolved organic matter cycling. Here, we construct a data-assimilating model of the open-ocean CH_4_ cycle to test which hypothesized mechanism is most consistent with the observed global CH_4_ distribution. We find that only linking methane production to phosphate (PO_4_) scarcity can explain the observed supersaturation pattern, which is highest in subtropical gyres where PO_4_ is in short supply. These findings suggest that CH_4_ release during PO_4_-limited cleavage of the organic compound methylphosphonate is the dominant production pathway in the open ocean. Because this process is confined to the stratified low latitude surface, it is uniquely suited to efficiently emit the CH_4_ it produces to the atmosphere (>90%), before the CH_4_ mixes to depth and undergoes oxidation (<10%). As predicted future ocean warming and stratification exacerbates PO_4_ scarcity over coming centuries, our model predicts that oxic CH_4_ production and the resulting CH_4_ emissions will increase up to twofold, contributing to a suite of positive feedback between climate warming and natural greenhouse gas sources.

## Significance

Methane is an important climate-warming gas, and its widespread emissions from well-oxygenated surface ocean waters (originally referred to as the “Marine Methane Paradox”) remains poorly understood. Using a global model assessed against field measurements, we evaluate the competing ideas for explaining oxic methane production. We identify phosphate scarcity as the primary environmental control, suggesting that methane is released when phosphate-starved microbes break down organic compounds to meet their phosphorus needs. This process is most active in subtropical ocean regions, where >90% of the methane produced escapes to the atmosphere before it is oxidized. Future climate warming will intensify ocean stratification and phosphate scarcity, likely strengthening this natural methane source to the atmosphere and contributing to amplification of the warming trend.

Methane (CH_4_) is a potent greenhouse gas with a 100-y global warming potential 28 to 34 times that of carbon dioxide (1). Curbing anthropogenic CH_4_ emissions is seen as a low hanging fruit for climate stabilization, but perturbation of natural CH_4_ sources by climate change would jeopardize this strategy (2). The global ocean contributes up to 10% of natural CH_4_ sources to the atmosphere, largely driven by diffusive emissions from supersaturated surface waters (3, 4). In coastal regions, CH_4_ supersaturation is generally attributed to seafloor sources, including hydrocarbon seeps and anoxic sediments (5, 6), whereas supersaturation in the surface open-ocean implies in-situ CH_4_ production in the well-oxygenated mixed layer (5, 7), which we refer to as oxic CH_4_ production hereafter. This phenomenon was originally referred to as the “Marine Methane Paradox” (8), given that methanogenesis is traditionally considered to be strictly anaerobic, and drives emissions of 1 to 2 Tg CH_4_ y^−1^ from the open-ocean surface (3). While this source is relatively small compared to the coastal ocean, the mechanisms responsible for oxic CH_4_ productions and their sensitivity to environmental change are particularly poorly understood aspects of the marine CH_4_ cycle.

Over the last two decades, several biological pathways have been proposed to explain the Marine Methane Paradox. Methane accumulation has been measured in cultures of ubiquitous phytoplankton clades, suggesting oxic CH_4_ production during photosynthesis (9–11). Methane may also be produced in the anoxic digestive tracts of zooplankton and fish and then egested into the oxic water column (12, 13). Other observations have connected CH_4_ production to the microbial cycling of three different dissolved organic matter (DOM) compounds found in the marine environment. First, the degradation pathway of dimethylsulfoniopropionate (DMSP), a phytoplankton metabolite, appears to yield CH_4_ if phosphate (PO_4_) is available for bacteria (14, 15). Conversely, the degradation of the reduced organic phosphorous compound methylphosphonate (MPn) produces CH_4_ under PO_4_-limited conditions (7, 16, 17). Finally, CH_4_ can be released as a by-product during the bacterial consumption of acetate (Ac), a photolysis product of other organic compounds (18, 19). While CH_4_ production by each of these pathways is supported by laboratory or local-scale field observations, their relative importance in explaining global scale open-ocean CH_4_ supersaturation remains unclear.

In this study, we exploit the signature that oxic production leaves in the ocean CH_4_ concentration ([CH_4_]) distribution. We use a global data-assimilating model to place constraints on the patterns and rates of biological CH_4_ production in the modern ocean, its environmental controls, and its sensitivity to future change (*Methods*).

## Results

### The Open-Ocean Methane Distribution.

**Fig. 1.**
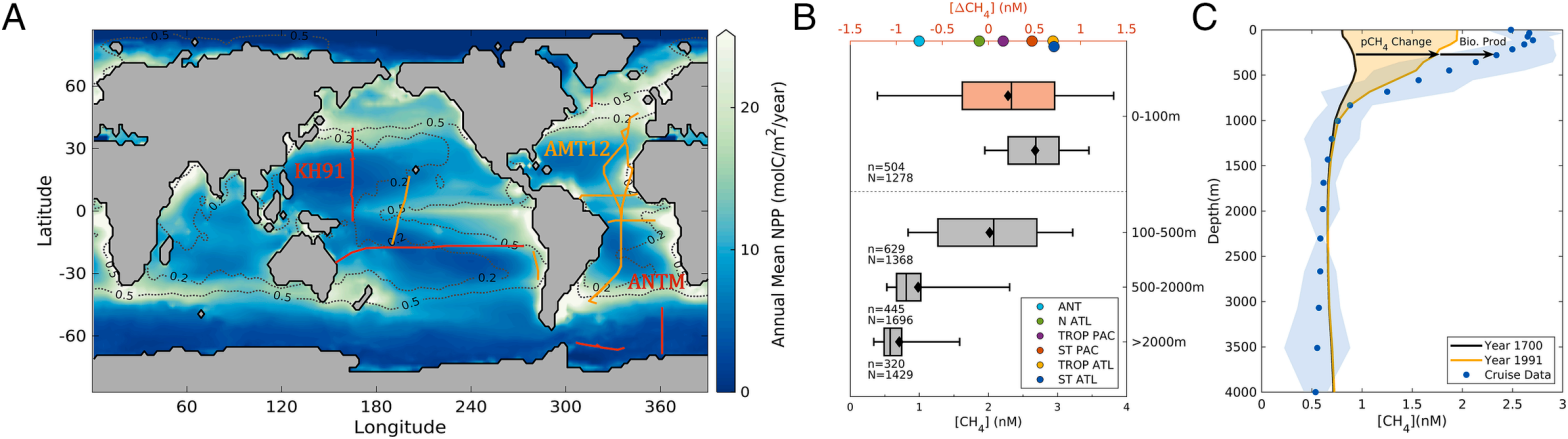
Data constraints and [CH_4_] distribution. (*A*) Locations of 11 cruise transects used as data constraints overlaid on climatological NPP (colored) and [PO_4_] (black contours). Orange lines indicate transects that resolve [CH_4_] to at least 500 m, whereas red lines resolve >1,000 m. (*B*) Boxplots summarize [CH_4_] from the 11 cruises in four depth intervals (whiskers are 5 to 95 percentiles, boxes are IQR, central lines and diamonds are median and mean, respectively, N is number of data points, and n is the number of model grid cells that contain data). Orange boxplot represents methane disequilibrium in the top 100 m, and colored scatters on the top x-axis represent mean surface supersaturation values for different ocean regions (legend). (*C*). Depth profiles comparing data from cruise KH-91 in the subtropical Pacific (blue dots), to model prediction without oxic CH_4_ production in year 1700 (black line) and year 1991 (year of cruise, yellow line). The blue shaded region indicates the cruise data uncertainty (1 SD), while yellow region depicts the [CH_4_] change attributed to increased atmospheric pCH_4_.

We focus our interpretation on field measurements from 11 cruise transects in the MEMENTO archive (20) that measured [CH_4_] with sufficient horizontal and vertical resolution to capture the interplay between key CH_4_ cycling processes: transport and mixing by the large-scale ocean circulation, equilibration with the atmosphere, aerobic oxidation, and modification near the surface by biological production (5) (Fig. 1*A*). Each transect extends to at least 500 m depth and therefore resolves [CH_4_] in subsurface waters that supply the surface, and five cruises resolve the whole water column down to >4,000 m. The transects span a range of marine environments, from polar to tropical, and cross sharp gradients in the properties that have been hypothesized to control oxic CH_4_ production (Fig. 1*A*).

The global CH_4_ distribution exhibits low and relatively uniform concentrations of 0.5 to 0.7 nM (interquartile range—IQR) in deep ocean waters (>2,000 m), which increase to 0.7 to 1.0 nM (IQR) in intermediate waters between 500 and 2,000 m (Fig. 1*B*). Concentrations further increase and reach maximum variance (1.3 to 2.7 nM, IQR) in subsurface waters between 100 and 500 m—a depth range that incorporates freshly ventilated downwelling water (high [CH_4_]) and aged upwelling water (low [CH_4_]). The highest [CH_4_] is observed in the upper 100 m (2.2 to 3.0 nM, IQR), where mixed layer waters are close to equilibrium with atmospheric pCH_4_ (Fig. 1*B*). However, the deviations from equilibrium (i.e., disequilibrium, [ΔCH_4_]) provide important clues about biological CH_4_ production. Most surface observations (62%) are supersaturated with respect to the atmosphere, especially in low latitudes (87% of observations). Averaging [ΔCH_4_] across large-scale provinces (21) reveals a distinct regional pattern (Fig. 1*B*), ranging from undersaturation in the Southern Ocean (ANT, −0.75 nM) and Subarctic North Atlantic (NATL, −0.10 nM), weak supersaturation in the Tropical Pacific (TPAC, 0.16 nM), modest supersaturation in the Subtropical Pacific (STPAC, 0.47 nM), and strongest supersaturation in the Tropical and Subtropical Atlantic (TATL = 0.70 nM, STATL = 0.71 nM respectively).

**Fig. 2.**
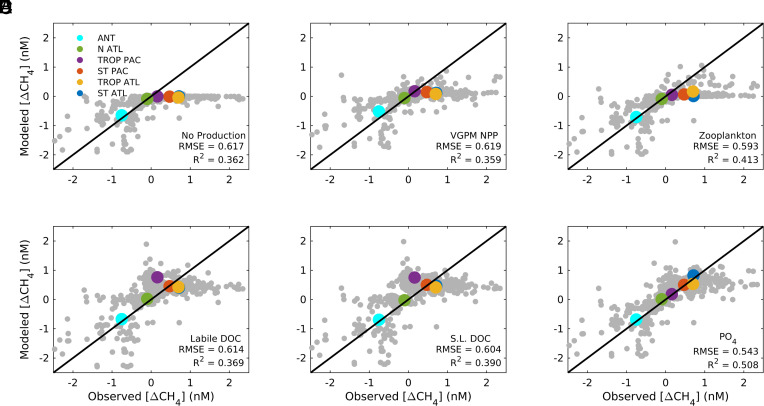
Model assessment of oxic CH_4_ production pathways. Predicted surface [ΔCH_4_] is compared to observations from the 11 cruises (gray dots) for model configurations with no oxic CH_4_ production (*A*), and those that link CH_4_ production to: (*B*) NPP; (*C*) Zooplankton metabolism; (*D*) Labile DOC (e.g., Ac); (*E*) Semilabile DOC (e.g., MPn); (*F*) PO_4_, which inhibits MPn degradation by C-P lyase. Colored dots average the modeled and observed [ΔCH_4_] into the same l regions as in Fig. 1*B*. Listed on each panel are the RMSE and R^2^ for all data (gray dots).

To interpret the observed open-ocean CH_4_ distribution, we developed a global model that simulates equilibration with observed historical pCH_4_ that increases from 706 to 1,832 ppm between 1700 and 2014 AD (22), transport by the large-scale circulation (23), and aerobic oxidation, which we model as a first-order process above a minimum [CH_4_] threshold (*Methods*). Following parameter optimization (*Methods*), a version of the model that omits oxic CH_4_ production was able to broadly reproduce observed [CH_4_] through the whole water column in high latitude regions (*SI Appendix*, Fig. S1), and in intermediate and deep waters (but not surface waters) in low latitudes (Fig. 1*C* and *SI Appendix*, Fig. S1). Averaged globally, surface ocean [CH_4_] increases from 1.4 nM in 1700 to 2.9 nM in 2014, as the ocean equilibrates with rising atmospheric pCH_4_ (*SI Appendix*, Fig. S2). This anthropogenic signal gradually penetrates the deep ocean, especially at high latitudes, raising the average deep ocean concentration by ~0.1 nM over the same period (*SI Appendix*, Fig. S2). Uptake of anthropogenic CH_4_ fully accounts for the vertical [CH_4_] gradient of ~2 nM observed in polar regions (*SI Appendix*, Fig. S1*B*), whereas in low latitudes the CH_4_ accumulation since 1700 only accounts for ~60% of the observed surface-deep gradient (Fig. 1*C* and *SI Appendix*, Fig. S1*A*). This is because the model poorly matches the surface [ΔCH_4_] pattern (R^2^ = 0.36), capturing undersaturation in high latitude regions but inaccurately predicting near-equilibrium conditions elsewhere (Fig. 2*A*)—a deficiency that must be attributed to the missing influence of oxic CH_4_ production.

### Oxic CH_4_ Production Pathways.

To determine which hypothesized CH_4_ production pathway can best reconcile our model with observed vertical and latitudinal gradients in [CH_4_] (Fig. 1*B*), we tested six parameterizations that link CH_4_ production to the biogeochemical properties thought to govern each pathway. Each parameterization includes three free parameters that we optimized (alongside the oxidation parameters) to minimize model-data misfit (*Methods*).

To test the hypothesis that methane is released during photosynthesis, we modeled CH_4_ production in the euphotic zone as proportional to satellite-derived Net Primary Production (NPP), and allowed the model to assign different production yields for NPP associated with three phytoplankton functional groups (24) (*Methods*). Following optimization, the model did not achieve a significantly better fit to surface [ΔCH_4_] than the version with no biological production (R^2^ = 0.36, Fig. 2*B*), and this result was not sensitive to the NPP product we selected, or to substituting gross primary production (GPP) for NPP in the parameterization (*SI Appendix*, Fig. S4 *C* and *D*). This is because linking CH_4_ production to NPP (or GPP) results in elevated CH_4_ production in productive high latitude regions and the Tropical Pacific, and weak production in subtropical regions, which act counter to the observed pattern of [ΔCH_4_]. To partially compensate, the model assigned very low CH_4_ yields to micro- and nano- plankton that dominate in mid-high latitudes and highest yield to picoplankton that dominate in the subtropics, which aligns with laboratory findings that cyanobacteria produce more methane than eukaryotes (9). This allows results in supersaturation up to ~0.15 nM in low latitudes (Fig. 2*B*)—an improvement over the version with no oxic production, but far short of observed [ΔCH_4_] in these regions.

We tested the hypothesis that CH_4_ is egested by zooplankton by linking methane production to zooplankton metabolism, which we modeled as a function of zooplankton biomass, oxygen, and temperature (*Methods* and *SI Appendix*, Fig. S3). The model achieved a marginal improvement in surface [∆CH_4_] (R^2^ = 0.41, Fig. 2*C*) compared to the NPP-based parameterization, but the regional biases remained unchanged. Because zooplankton biomass exhibits a similar distribution to NPP (25), the optimization again selected for low overall CH_4_ production to avoid unrealistically high [ΔCH_4_] in high latitudes and the Tropical Pacific, which in turn keeps [ΔCH_4_] too low in the subtropics. The model partially compensates by assigning a strong temperature sensitivity to zooplankton metabolism, which helps concentrate CH_4_ production in low latitudes. To achieve this, a Q_10_ coefficient of ~4 was selected (*Methods*), which is beyond the typical range of 2 to 3 for biological kinetics (26), casting further doubt on this mechanism.

We investigated the hypothesized production of CH_4_ during microbial degradation of dissolved organic substrates by linking oxic CH_4_ production to the distributions of dimethylsufide (DMS), labile DOM, and semilabile DOM, obtained from data-driven modeling (27, 28) (*Methods*). The DMS-based model represents CH_4_ production during DMSP degradation, since DMS is a byproduct of DMSP and the two share similar distributions, but with far more observations for DMS (*SI Appendix*, Fig. S3*A*) (27). This model yielded a fit to surface [∆CH_4_] that was indistinguishable from the version with no biological CH_4_ production (*SI Appendix*, Fig. S4), because the DMS distribution is similar to NPP, resulting in the same latitudinal biases. The parameterization based on labile DOM represents CH_4_ production during Acetate (Ac) consumption, since Ac is an organic compound with an observed turnover time of days to weeks, like the labile DOM pool (19, 28). This model shifts the pattern of CH_4_ production toward low latitudes and achieves higher supersaturation compared to the NPP-based parameterization (Fig. 2*D*), because labile DOM does not accumulate in productive high latitude regions (*SI Appendix*, Fig. S3*C*) due to deep mixing (28). However, it strongly overpredicts [ΔCH_4_] in the tropical Pacific and underpredicts [ΔCH_4_] in the oligotrophic subtropical Atlantic (R^2^ = 0.37). The parameterization based on semilabile DOM represents substrate-limited CH_4_ production during MPn cycling, since MPn is a ubiquitous component of the semilabile organic pool (17) that renders CH_4_ when metabolized by the C-P lyase enzyme complex (29). Compared to the labile DOM parameterization, this model partially shifts CH_4_ production from productive upwelling regions toward downstream regions of DOM accumulation (*SI Appendix*, Fig. S3*D*), which improves the [ΔCH_4_] prediction (R^2^ = 0.39), but does not rectify biases in the tropical Pacific and subtropical Atlantic (Fig. 2*E*).

**Fig. 3.**
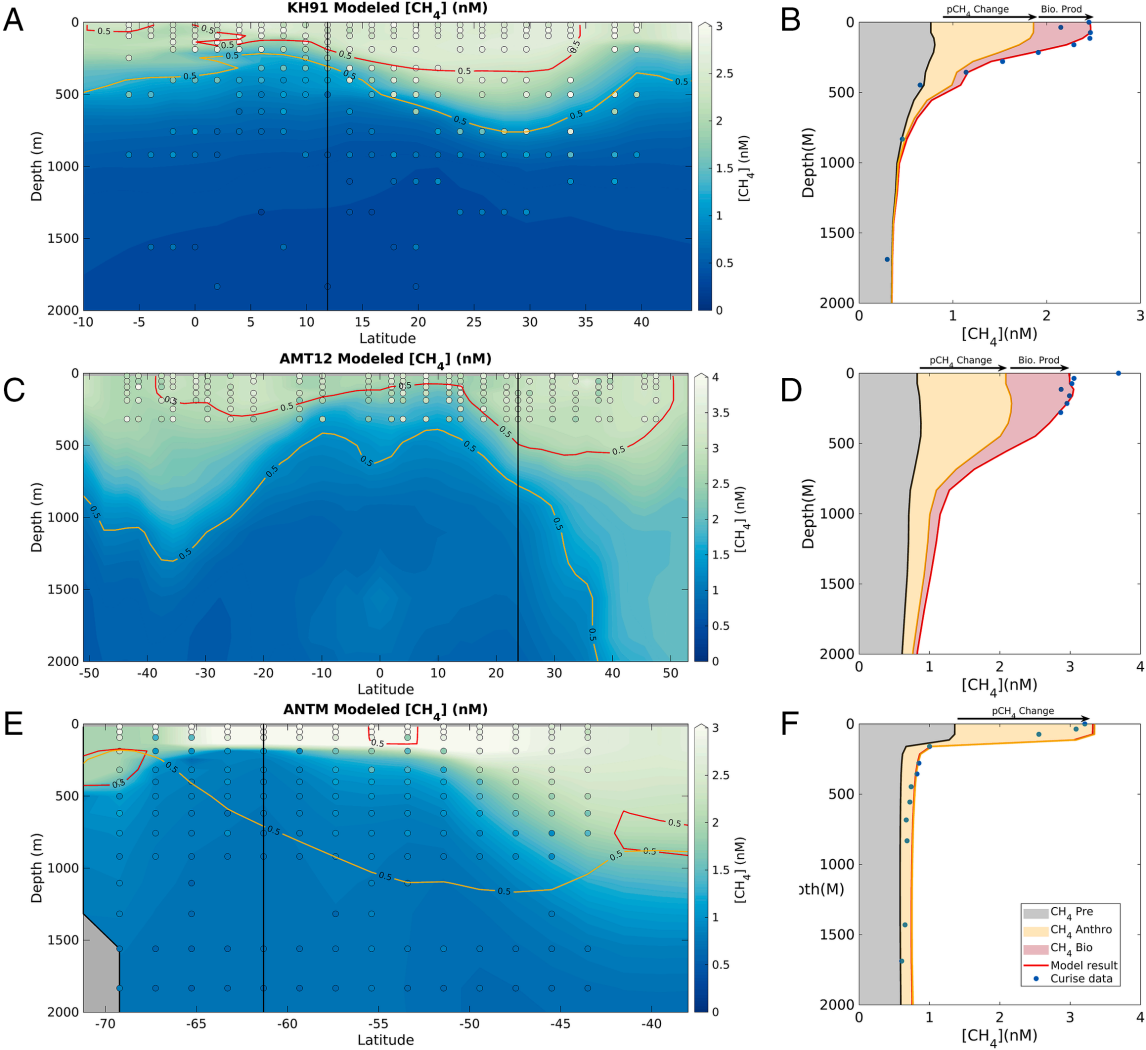
Methane distribution and component interpretation. Predicted [CH_4_] from model with CH_4_ production linked to PO_4_, compared to observations from representative transects: (*A* and *B*) AMT 12 in the Atlantic Ocean; (*C* and *D*) KH91 in the Pacific Ocean; (*E* and *F*) ANTXV/4 M in the Southern Ocean (see Fig. 1*A* for locations). The *Left* panels show model-predicted [CH_4_] along the whole transect (background color), with observations overlaid (colored dots); *Right* panels compare the model profile (red line) to observations (blue dots) at locations marked on the transects by a thick black line. The orange and red contours in *Left* panels represent the concentration of anthropogenic (CH_4,anthro_) and biologically produced (CH_4,bio_) CH_4_ components, at 0.5 nM intervals. In the *Right* panels, modeled profile is broken into the CH_4,bio_ (red shading), CH_4,anthro_ (orange shading), and CH_4,pre_ (gray shading).

An additional control on MPn cycling is the availability of ambient PO_4_, since microbes cleave MPn via C-P lyase to access phosphorus when PO_4_ is scarce (7, 17), whereas other degradation pathways are favored when PO_4_ is replete (30). To represent this, we tested a final model configuration in which CH_4_ production is parameterized as an inverse function of [PO_4_], using a global distribution corrected for biases at low-[PO_4_] (31) (*Methods*). This model reproduced the surface [ΔCH_4_] pattern significantly better than any other configuration (R^2^ = 0.51), because it restricts CH_4_ production to oligotrophic low latitude regions while allowing the PO_4_-replete Tropical Pacific to remain close to equilibrium as observed (Fig. 2*F*). It is the only model able to capture the higher observed [ΔCH_4_] in the subtropical Atlantic relative to the Pacific (Fig. 2*F*), and the observed increase in [ΔCH_4_] from the southern to northern subtropical gyres in both basins (Fig. 3 *A*–*D*). These patterns can only be explained by linking CH_4_ production to PO_4_ scarcity, rather than oligotrophy in general, since nitrate (another other key nutrient) is uniformly low throughout the subtropics (32). Recent measurements across the subtropical North Atlantic Ocean (not used during model optimization) found some of the highest surface [CH_4_] ever observed in the open ocean (3 to 8 nM) (33). Again, the model linking CH_4_ production to PO_4_ scarcity was the only one to predict [CH_4_] within the observed range along this cruise track in the ocean’s most PO_4_-stressed biome (31).

The relative skill of the PO_4_-based model suggests that the large-scale pattern of [ΔCH_4_] in the open ocean is best explained by CH_4_ production during MPn cleavage, predominantly controlled by PO_4_ scarcity rather than MPn availability. In fact, a model version that links CH_4_ production to both PO_4_ and semilabile DOM (*Methods*), to reflect the influence of both factors on MPn cleavage, does not make a discernable improvement (R^2^ = 0.52) despite employing more free parameters. We cannot rule out other production pathways making smaller contributions to CH_4_ supersaturation, although an additional model version that combines the photosynthetic and PO_4_-inhibited parameterizations (*Methods*) makes no improvement to [ΔCH_4_] (R^2^ = 0.5). The model with oxic CH_4_ production linked only to PO_4_ is therefore adopted for further analysis in this study.

### Components of Open-Ocean Methane.

In addition to explaining the [ΔCH_4_] pattern in the surface ocean (Fig. 2), our model also skillfully reproduces CH_4_ concentrations globally across all depths (R^2^ = 0.78; Fig. 3 and *SI Appendix*, Figs. S5 and S6), allowing us to build a mechanistic interpretation of the global ocean methane distribution. We characterize this as the sum of three components depending on the CH_4_ source, which we isolated through further simulations (*Methods*): i) CH_4_ originating from air–sea equilibration with preindustrial atmospheric pCH_4_ levels as of 1700 A.D. (CH_4,pre_), ii) CH_4_ accumulated since 1700 due to equilibration with elevated atmospheric pCH_4_ resulting from anthropogenic emissions (CH_4,anthro_), and iii) CH_4_ produced biologically in the oxygenated surface ocean (CH_4,bio_).

Preindustrial CH_4_ (CH_4,pre_) has a surface distribution controlled by temperature-dependent solubility, ranging from ~0.8 nM in warm tropical waters (Fig. 3 *A* and *C*) to >1.3 nM in the Southern Ocean (Fig. 3*E*), and an interior distribution controlled by the relative timescales of ocean circulation vs. oxidation. Given the oxidation timescale of 172 y selected by the optimization, CH_4,pre_ converges toward the lower oxidation threshold (~0.33 nM) in water masses older than 500 y, e.g., below 1,000 m in the North Pacific Ocean (Fig. 3*A*). In contrast, CH_4,pre_ remains elevated above the oxidation threshold below 1,000 m in the North Atlantic and Southern Ocean, due to ventilation of deep and intermediate waters on timescales <500 y. Overall, our model indicates that equilibration with the preindustrial atmosphere accounts for the vast majority (87.5%) of dissolved CH_4_ in the deep ocean (>2,000 m), but only approximately half of the CH_4_ in the intermediate (100 to 2,000 m) and surface (<100 m) layers (58.4% and 47.7% respectively; *SI Appendix*, Fig. S7). Therefore, CH_4_,_pre_ exhibits a weak vertical gradient compared to observed [CH_4_] (Fig. 3 *B*, *D*, and *F* and *SI Appendix*, Fig. S6), highlighting the importance of the other two components in controlling the global depth structure of [CH_4_].

The surface distribution of anthropogenic CH_4_ (CH_4,anthro_) is controlled by solubility and increases through time in lockstep with atmospheric pCH_4_ (*SI Appendix*, Fig. S2). In the 1990s, when most of the measurements used in this study were collected, its contribution to observed surface [CH_4_] (41.7% globally) already rivaled that of CH_4,pre_ (Fig. 3 *B*, *D*, and *F* and *SI Appendix*, Fig. S6), but its relative contribution declines rapidly with depth. Subsurface penetration of CH_4,anthro_ is restricted to waters that were ventilated since significant growth in atmospheric pCH_4_ (~100 y, *SI Appendix*, Fig. S2), which limits it to the upper 500 m influenced by wind-driven downwelling in low latitudes (Fig. 3*B*). Only in regions of rapid deepwater formation like the North Atlantic does CH_4,anthro_ make a significant contribution to observed [CH_4_] at depths of 1,000 m and below (Fig. 3*D*), and globally it accounts for only 12.5% of deep ocean methane (*SI Appendix*, Fig. S7).

Of the three components, CH_4_ produced biologically in surface waters (CH_4,bio_) has the most heterogeneous distribution, both geographically and vertically. In the surface, CH_4,bio_ is largely confined to low-PO_4_ subtropical regions, where it contributes ~25% of observed [CH_4_] in the subtropical Pacific (Fig. 3*A*) and >30% in the subtropical Atlantic (Fig. 3*C* and *SI Appendix*, Fig. S6 *E* and *F*). Since very little CH_4,bio_ is produced in mid- and high-latitude regions that ventilate the ocean interior, its subsurface distribution is limited to waters ventilated by low-latitude downwelling. It therefore penetrates only to depths of ~500 m in the centers of subtropical gyres where downwelling is strongest, and makes minor contributions to the intermediate and deep CH_4_ inventories (1.8% and <0.10% respectively; *SI Appendix*, Fig. S7). While the link between oxic methane production and PO_4_-scarcity limits its contribution to the global ocean CH_4_ inventory, it amplifies the sea-air emissions driven by the process, by restricting CH_4_ production to waters that equilibrate with the atmosphere faster than they mix with the ocean interior.

### The Fate of Biologically Produced Methane.

**Fig. 4.**
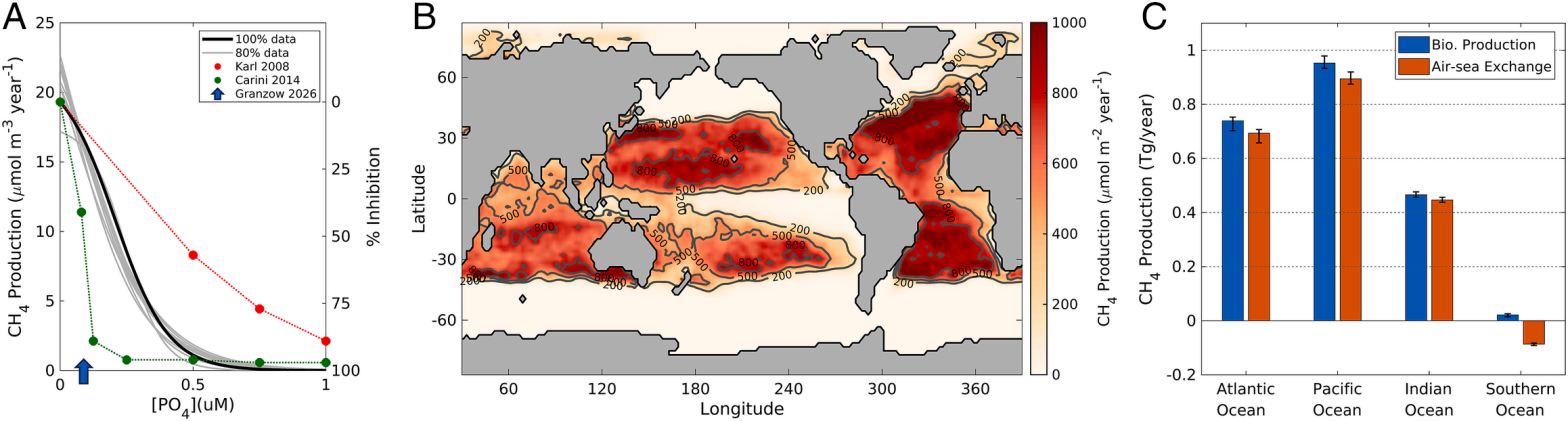
Oxic CH_4_ production distribution and budget. (*A*) Oxic CH_4_ production rate as a function of [PO_4_]. The black line is our model-derived parameterization when optimized with 100% of the data, while the gray lines are optimized with random samples of 80% of the data. Red and green dots are measurements from seawater incubations (7) and single-species cultures (16), plotted as % inhibition of CH_4_ production relative to rates when PO_4_ is absent (right y-axis). The blue arrow shows the PO_4_ threshold for C-P lyase activity in a bacterial monoculture (35). (*B*) Depth-integrated oxic CH_4_ production rate, from optimization using 100% of the data. (*C*) Basin-integrated rates of oxic CH_4_ production and sea-air exchange. Bar centers from optimization using 100% of data, error bars are 1 SD from the optimizations using 80% of the data.

To investigate the fate of CH_4_ originating from oxic biological production, we constructed a surface CH_4_ budget from our model, and propagated uncertainty with a bootstrapping approach in which the model was reoptimized using random data subsets. All optimizations converged on similar relationships between CH_4_ production and [PO_4_], reaching a theoretical maximum rate of ~20 µmolCH_4_/m3/year when PO_4_ is absent then rapidly decline to 6% of the maximum (i.e., 94% inhibition) at 0.5 µM [PO_4_] (Fig. 4*A*). This degree of inhibition is somewhat stronger than observed in seawater incubations, where CH_4_ production persisted up to ~0.4 µM PO_4_ in unamended samples (34), or was only ~60% inhibited by addition of 0.5 µM PO_4_ (7) (Fig. 4*A*). However, stronger inhibition has been observed in bacteria monocultures, where CH_4_ production was ~95% inhibited by 0.25 µM PO_4_ (16), and C-P lyase activity was not detected above ~0.1 µM PO_4_ (35). Our model-derived relationship lies between the species-level (from monocultures) and community-level (from seawater incubations) constraints (Fig. 4*A*) and motivates further work to identify the suite of organisms that cleave MPn in the ocean, and the PO_4_ sensitivity of this pathway.

Our global model predicts a distribution of oxic methane production that is largely confined within the 0.5 µM climatological PO_4_ contour (Fig. 4*B*), and resembles the distribution of C-P lyase enzyme expression in marine microorganisms (30). The process is limited to low latitude regions and peaks in the centers of the subtropical gyres, especially the North Atlantic Subtropical Gyre where PO_4_ reaches its global minimum of ~0.012 µM according to high precision measurements (Fig. 4*B*) (31). Integrated across ocean basins (Fig. 4*C*), the Pacific Ocean in responsible for the most oxic CH_4_ production (0.96 ± 0.02 Tg/y) due to the vast area of the Pacific subtropical gyres, followed by the Atlantic Ocean (0.73 ± 0.03 Tg/y) and then the Indian Ocean (0.47 ± 0.01 Tg/y). The Southern Ocean stands out as a region of negligible CH_4_ production (0.02 ± 0.01 Tg/y), given that surface [PO_4_] in this basin rarely drops below the 0.5 µM level required for CH_4_ production.

There are three potential fates for CH_4_ produced biologically in the surface ocean: emission to the atmosphere, aerobic oxidation in surface waters, or transport into the ocean interior followed by oxidation at depth. In our observationally constrained model, sea-air CH_4_ emissions sum to 0.90 ± 0.02 Tg/y, 0.68 ± 0.03 Tg/y, and 0.45 ± 0.01 Tg/y in the Pacific, Atlantic, and Indian Oceans respectively, amounting to >90% of oxic methane production rate in each basin (Fig. 4*C*). In the Southern Ocean, the upwelling of CH_4_-depleted deep water and the lack of oxic CH_4_ production results in surface CH_4_ undersaturation, driving the net uptake of 0.09 ± 0.01 Tg/y, making this region a sink for atmospheric methane.

Summing globally, our model estimates that 2.15 ± 0.06 Tg CH_4_/y is produced biologically in the surface ocean, of which 2.03 ± 0.06 Tg/y is emitted to the atmosphere (Fig. 4*C*), aligning with the upper end of the range of previous emissions estimates (3, 4). Emission to the atmosphere is therefore the dominant fate for biologically produced CH_4_, which is explained by the distribution of CH_4_ production and the timescale of the alternative sink processes. Given characteristic gas exchange velocities of 1 to 10 m/d (36), most mixed layers fully equilibrate with the atmosphere on timescales shorter than 1 y. Because the open-ocean oxidation timescale diagnosed by our model is much longer than this, oxidation within the mixed layer is a negligible sink, removing only 2.21% of biologically produced CH_4_. In high-latitude regions of deep seasonal mixing, physical export of CH_4_ to the ocean interior could occur on rapid timescales that rival air–sea equilibration. However, the requirement for PO_4_ scarcity means that oxic CH_4_ production is excluded from these regions and focused in stratified subtropical gyres where physical export occurs only through sluggish downwelling. The mechanism and distribution of oxic production are therefore uniquely favorable for outgassing of CH_4_ to the atmosphere.

## Discussion

**Fig. 5.**
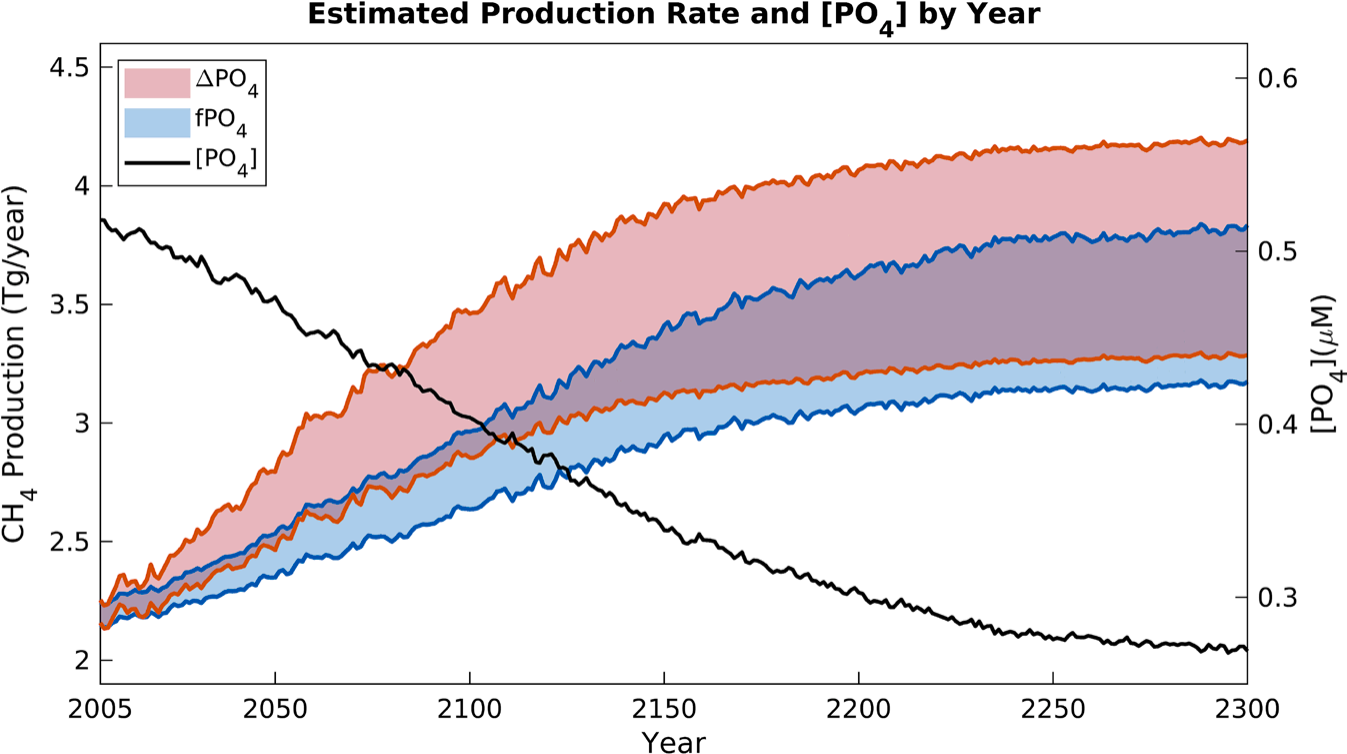
Oxic CH_4_ production in the future ocean. Red and blue envelopes (corresponding to the left y-axis) illustrate how the predicted globally integrated oxic CH_4_ production rate will change up to the year 2300 under two different scenarios for future PO_4_ depletion. Red envelope applies the spatially varying absolute change in [PO_4_] from ref. 37 to the observed PO_4_ distribution used in our CH_4_ cycle model, whereas blue applies the relative [PO_4_] change (*Methods*). Black line (corresponding to the right y-axis) shows the predicted change in global-mean surface [PO_4_].

Our model optimizations found that MPn cleavage in PO_4_-starved waters is the oxic CH_4_ production mechanism most consistent with observed [CH_4_], and that the distribution of this process promotes emission (rather than export and oxidation) of CH_4_. This raises the potential for feedbacks between climate-induced changes in the PO_4_ cycle and marine emissions of the greenhouse gas CH_4_. Biogeochemical simulations under the high-emissions SSP5-8.5 future climate scenario predict that ocean stratification and high latitude nutrient utilization will reshape ocean nutrient distributions and stifle the nutrient supply to low latitude surface waters (37, 38). A simulation of marine biogeochemistry that extends to 2300 predicts that average surface [PO_4_] in low latitudes surface waters will decrease by ~25% from ~0.61 µM in 2005 to ~0.46 µM by the year 2300 (37) (Fig. 5 and *SI Appendix*, Fig. S8 *A* and *B*).

To estimate the first-order impact of these changes on marine CH_4_ production, we imposed predicted future changes in PO_4_ (37) on our model parameterization (*Methods*). We explored two scenarios, imposing either the absolute or relative future changes in PO_4_, and in both cases we propagated uncertainty associated with our parameterization (Fig. 4*A*). Our model predicts that as PO_4_-scarcity becomes more widespread and intense in the surface ocean, marine oxic CH_4_ production will increase to 2.9 to 3.5 Tg/y by 2100 and 3.3 to 4.2 Tg/y by 2300, representing a 52 to 86% increase relative to 2005 (Fig. 5 and *SI Appendix*, Fig. S8 *C*–*F*). Climate-driven changes in nutrient cycling therefore have significant potential to perturb the open-ocean methane cycle and marine emissions of this important greenhouse gas.

Although an additional source of ~2 Tg CH_4_/y from the ocean is relatively minor compared to direct anthropogenic emissions [~300 Tg/y, (4)], most future climate scenarios assume that anthropogenic sources will peak and then decline during the next few centuries (39). Our work therefore highlights a climate feedback that could partially offset this trend, and contributes to a growing suite of feedback loops that have been recognized between climate warming and natural CH_4_ sources to the atmosphere (2, 40). These include other perturbations to the marine CH_4_ cycle, such as intensification of anaerobic methanogenesis in sediments due to warming (41) and deoxygenation (42), expansion of suboxic waters (43) that may stifle CH_4_ removal by oxidation (44), and destabilization of hydrates in high latitude basins (45), where CH_4_ could be released shallow enough to evade oxidation before release to the atmosphere (46). These potential feedbacks motivate further work to better characterize the rates and climate sensitivities of marine CH_4_ sources and sinks.

While our study has identified PO_4_ scarcity as the primary driver of oxic CH_4_ production in the open ocean, our understanding of the MPn cleavage pathway and its inhibition by PO_4_ across diverse microbial groups is still in its infancy. Furthermore, our modeling approach and the available data constraints are insufficient to diagnose additional controls on CH_4_ production rates in P-limited environments. These could include production rates of MPn by cyanobacteria (33), advective MPn supply along gyre margins from upwelling regions where PO_4_ inhibits its degradation, and the activity of the C-P lyase complex, which can be limited by iron (47). Additional fieldwork, experiments, and modeling are required to unravel how these factors interact with PO_4_ availability to modulate oxic CH_4_ production and shape its sensitivity to environmental change.

## Methods

We constructed a three-dimensional model of the open-ocean methane cycle that assimilates field measurements to tune uncertain biogeochemical process and test hypothesized mechanisms for oxic CH_4_ production.

### Marine Methane Model.

In our model, the three-dimensional temporal evolution of methane concentration (denoted *C,* in nanomolar units) is represented by the differential equation:

[1] $$\frac{\mathrm{dC}}{\mathrm{dt}}=AC+J_{\mathrm{prod}}+J_{gas-ex}-J_{\mathrm{ox}}.$$

Here, AC represents the physical transport and mixing of methane, resolved using the transport matrix method (48). **A** is the transport matrix (with units y^−1^) derived from the Ocean Circulation Inverse Model, OCIMv2 (23). All other terms (*J*) on the right-hand side ofEq. **1** represent CH_4_ sources and sinks. $J_{\mathrm{prod}}$ is the oxic methane production rate, parameterized differently for each hypothesized CH_4_ production pathway (*Methods*), and $J_{\mathrm{gx}}$, is air–sea gas exchange, proportional to the difference between saturation and dissolved [CH_4_]:

[2] $$J_{\mathrm{gx}}=Q_{\mathrm{gx}}*(S_{CH4}-C).$$

Here, $S_{CH4}$ represents the saturation concentration, calculated as

[3] $$S_{CH4}=X_{CH4}*P*K.$$

Here, $X_{CH4}$ is the atmospheric methane partial pressure, which increases as a function of time, *P* is the sea-level air pressure, and *K* is the methane solubility calculated from sea surface temperature and salinity (49). InEq. **2**, $Q_{\mathrm{gx}}$ is a gas exchange operator (with units y^−1^) defined (for surface grid cells, and zero elsewhere) as

[4] $$Q_{\mathrm{gx}}=\frac{\left(1-f_{\mathrm{ice}}\right)*k_{w}}{d}.$$

Here, $f_{\mathrm{ice}}$ is the fractional sea-ice coverage of surface ocean grid cells, *d* is the thickness of the top layer in the model (36.14 m), and *k_w_* is the piston velocity following (36):

[5] $$k_{w}=0.251*U^{2}*{\left(\frac{\mathrm{Sc}}{660}\right)}^{-0.5}.$$

In Eq. **5**, U is the average wind speed at 10 m above the sea surface and Sc is the Schmidt number that can be calculated as a function of sea surface temperature. In Eq. **1**, $J_{\mathrm{ox}}$ represents aerobic methane oxidation which we model as first-order loss constrained by a threshold concentration:

[6] $$J_{\mathrm{ox}}=\min\left(0,k_{\mathrm{ox}}*\left(C-C_{\mathrm{crit}}\right)\right).$$

Here, $k_{\mathrm{ox}}$ is the aerobic oxidation rate constant (with units y^−1^), and *C_crit_* is a threshold CH_4_ concentration beneath which oxidation ceases. While methane oxidation is typically considered a first-order process, we incorporated the threshold effect to reflect the fact that microbially mediated reactions tend to deplete substrates to a break-even level beneath which a microbial community cannot be sustained. This is supported by the observation that [CH_4_] never drops below ~0.3 nM, even in the oldest deep ocean waters (Fig. 1*B*). We tested alternative formulations of $J_{\mathrm{ox}}$, including linking $k_{\mathrm{ox}}$ to temperature and oxygen and representing oxidation as a higher-order function of [CH_4_]. None of these approaches improved the model fit to observations, even while adding new free parameters to the model.

### Data Input and Constraints.

We used the Cross-Calibrated Multi-Platform Wind Vector Analysis (CCMP) climatology product (50) to define *U* in Eq. **5**, the NCEP reanalysis product (51) to define *P* in Eq. **6**, the NOAA Optimum Interpolation V2 product (52) to define *f_ice_* in Eq. **4**, and the MIMOC mixed layer product (53) for temperature and salinity. Annual-mean climatologies for each dataset were linearly interpolated to the OCIM grid. The historical record of annual-mean atmospheric methane mixing ratio is taken from the CMIP6 forcing file (22) which merges ice core reconstructions with direct measurements. We defined the 1700 A.D., mixing ratio of 706 ppb as a preindustrial steady state for rapid model spin-up, given that atmospheric methane changes before this date were negligible.

Our model uses a subset of field measurements from the MEMENTO (Marine Methane and Nitrous Oxide) archive (20) to assess model performance and tune uncertain parameters. Three selection criteria were used. First, we focused on vertical profiles of [CH_4_] measured from discrete vial samples, and excluded cruises with surface-only data from underway equilibrators. Equilibrators are subject to bias associated with headspace venting, which may be particularly problematic at the low concentrations characteristic of the open ocean (54, 55), and measurements at the air–sea interface are subject to short timescale variability associated with winds, so are less representative than vial measurements collected through the whole mixed layer. Second, we selected cruises with sufficient vertical sampling (at least 500 m deep) to capture the methane concentrations supplied to the surface via mixing and upwelling. To find the pattern of oxic CH_4_ production most consistent with observed mixed layer [CH_4_], our model must accurately account for the physical supply from below. Finally, we excluded cruises that cross Oxygen Minimum Zones (OMZs, where O_2_ < 5 μM), which host complex microbial communities that can produce or consume methane through pathways not typically active in oxic waters (5, 56) and that are not resolved in our model. These criteria yielded 11 cruises that capture the global ocean [CH_4_] distribution across a broad range of biogeochemical environments, from polar latitudes to subtropical gyres.

### Model Solution.

The model solution comprises two stages. First, the model is solved for the preindustrial ocean CH_4_ distribution at 1700 A.D. by setting and $X_{CH4}$ = 706 ppb in Eq. **3** and solving Eq. **1** for steady state using Newton’s method (facilitated by the Transport Matrix formulation). In the second stage of the solution, the system is integrated forward in time from the year 1700 to 2014, to resolve the temporal evolution of atmospheric pCH_4_ and its impact on oceanic [CH_4_]. We use a hybrid timestep scheme comprising a Crank-Nicolson method and Euler method developed for matrix models with linear and nonlinear terms (57, 58). Model output is recorded at 10-y intervals from 1700 to 1990, and at 1-y intervals from 1991 to 2014.

### Oxic CH_4_ Production Parameterizations.

In Eq. **1**, $J_{\mathrm{prod}}$ (μmol m^−3^ y^−1^ units) is parameterized differently for each hypothesized oxic CH_4_ production pathway. Each parameterization contains three free parameters to place them on equal footing, and confines CH_4_ production to surface layers of the ocean consistent with the production mechanism. For organic matter cycling, *J_prod_* occurs above the wintertime mixed layer depth (*z_ML_*) (53), since labile organic matter pools and bacterial biomass are strongly depleted beneath the pycnocline (28). For photosynthetic production, *J_prod_* occurs above the climatological euphotic zone depth (*z_eu_*) (59). For zooplankton egestion, *J_prod_* occurs between the surface and the characteristic diel vertical migration depth (*z_DVM_*) of migrating fauna (60).

#### Photosynthetic CH_4_ production.

Based on recent evidence for CH_4_ production during photosynthesis by diverse phytoplankton clades (9–11) we parameterized *J_prod_* as proportional to NPP, while allowing for divergent CH_4_ yields between three broad phytoplankton categories—microplankton (m), nanoplankton (n), and picoplankton (p):

[7] $$J_{\mathrm{prod}}=(a_{m}f_{m}+a_{n}f_{n}+a_{p}f_{p})\;NPP\;(z<z_{\mathrm{eu}}).$$

Here, climatological *NPP* is taken from the VGPM satellite algorithm (25), the f parameters represent the fraction of NPP attributed to each phytoplankton group estimated from chlorophyll-a (61), and a is the CH_4_ yield per unit biomass production for each group. Additional model configurations were tested in which chlorophyll-based NPP from VGPM was replaced with carbon-based NPP from the CbPM algorithm (62), or GPP from a machine learning product (63), but all configurations yielded similar results (*SI Appendix*, Fig. S4).

#### Zooplankton egestion.

Zooplankton can egest CH_4_ from their anoxic digestive tracts into the water column, but relatively little is known about the rates and distribution of the process, or whether it is clade-specific (12, 13). Our model therefore takes a simple approach and assumes that methane egestion is proportional to zooplankton metabolism, which in turn scales with their biomass (*B_Z_*), modulated by ambient oxygen concentration (*O_2_*) and temperature (*T*) (64):

[8] $$J_{\mathrm{prod}}=a_{\mathrm{zoo}}*B_{z}*{Q_{10}}^{\frac{T-T_{\mathrm{ref}}}{10}}*\frac{O_{2}}{O_{2}+{\mathrm{kO}}_{2}}\;(z<z_{\mathrm{DVM}}).$$

Here, $a_{\mathrm{zoo}}$ is the CH_4_ production rate per unit biomass at a reference temperature (*T_ref_* = 20 °C), and $Q_{10}$ and ${\mathrm{kO}}_{2}$ are temperature-sensitivity and oxygen half-saturation constants, respectively. We follow (65) and estimate $B_{z}$ globally using a log–log relationship between zooplankton biomass data from the COPEPOD archive (66) and chlorophyll-a from the MODIS satellite program. In Eq. **8**, temperature and oxygen distributions are taken from World Ocean Atlas 2018 (WOA18) (67, 68).

#### Substrate-limited organic matter cycling.

We modeled the production of CH_4_ during microbial organic matter cycling by assuming that the decomposition of an organic substrate is limited by the substrate concentration (*S*) and the biomass of the microbial population. Assuming the population is in quasi state with the substrate (*SI Appendix*, *Supplementary Methods*) this yields:

[9] $$J_{\mathrm{prod}}=a_{s}\frac{S}{S+K_{S}}\left(\frac{S}{S+K_{S}}-b_{s}\right).$$

Here, *a_s_* defines a theoretical maximum production rate and $b_{s}$ introduces a threshold effect, where production stops at $S<b_{s}K_{s}/\left(1-b_{s}\right)$, i.e., the break-even *S* concentration for the community. This provides a flexible parameterization that can take a range of functional forms for different selections of the free parameters ($a_{s}$, $b_{s}$, $K_{s}$). We applied Eq. **11** to three different organic substrate distributions to represent three different proposed CH_4_ production pathways. To represent CH_4_ production during DMSP cycling we use the global DMS distribution from a machine learning product that extrapolates field measurements (27). As proxies for the Ac and MPn distributions, we use the labile and semilabile DOC distributions from a mechanistic model that assimilates DOC observations and additional tracer data (28).

#### Phosphate-limited MPn degradation.

While microbes can decompose MPn via C-P lyase as a phosphorus source, they preferentially utilize dissolved phosphate (PO_4_), and therefore this CH_4_ production pathway is inhibited at high ambient [PO_4_] (7). We adopt the following parameterization for PO_4_-inhibited CH_4_ production related to MPn cleavage:

[10] $$J_{\mathrm{prod}}=-a_{0}\left(0.5\times \tanh\left(\frac{\left(\left[{\mathrm{PO}}_{4}\right]-P_{\mathrm{crit}}\right)}{w}\right)-0.5\right).$$

Here, *a_0_* is the theoretical maximum rate when PO_4_ is depleted, *P_crit_* denotes a critical [PO_4_] concentration at the center of the hyperbolic tangent function, and the width-factor *w* determines how sharply *J_prod_* declines as a function of [PO_4_]. This functional form is selected for flexibility, given that different parameter choices can result in near-linear, exponential, or step-function relationships. Here, the [PO_4_] distribution is taken from WOA18 (32) then corrected for the tendency of this product to overestimate [PO_4_] at very low concentrations (31) (*SI Appendix*, *Supplementary Methods*), although our results are not sensitive to this correction (*SI Appendix*, Fig. S4*B*).

### Optimization.

We used a gradient-search algorithm (fminserach in MATLAB) to seek the free parameter values that minimize the misfit between model-predicted and observed [CH_4_] distributions, allowing us to identify the most plausible parameterization for *J_prod_*. In each model configuration, the CH_4_ oxidation parameters *k_ox_* and *C_crit_* (in Eq. **4** Eq. **6**) are optimized, along with three additional parameters in each formulation of *J_prod_* (*a_m_*, *a_n_*, and *a_p_* in Eq. **7**; *a_zoo_*, *Q_10_*, and *K_O2_* in Eq. **8**; *a_S_*, *K_S_*, and *b_S_* in the three different applications of Eq. **9**; and *a_0_*, *P_crit_*, and *w* in Eq. **10**). Model solutions are assessed against cruise data by interpolating the prediction for the year of each cruise to the exact location of each [CH_4_] measurement. The RMSE for each cruise transect (*i*) is calculated, and the overall model-data misfit is quantified by a cost function, defined as the weighted sum of across all transects:

[11] $$\mathrm{cost}=\sum_{i=1}^{k}{\mathrm{RMSE}}_{i}w_{i}.$$

Here, w_i_ is the weighting factor for each transect, based on the number of datapoints it comprises, and *k* = 11 is the total number of transects. Typically, ~200 solutions of the forward model are required for the algorithm to converge on an optimum parameter set that minimizes the cost. Initial selections and optimized values for each parameter are provided in *SI Appendix*, Table S1. Having identified the model configuration that best replicates the global CH_4_ distribution (which uses Eq. **10** for *J_prod_*), we estimate uncertainty in the model parameters by repeating the optimization process 12 times using 80% of the data constraints each time (Fig. 4*A*). This uncertainty is propagated in the global CH_4_ budget (Fig. 4*C*) and future predictions (Fig. 5).

### Component Analysis.

We divided modeled [CH_4_] into three components according to its source by rerunning our best-fitting model with oxic CH_4_ production disabled (*J_prod_* = 0), while holding other parameters at their optimized values. The initial steady-state CH_4_ distribution in this configuration represents CH_4_ that originates from equilibration with the preindustrial atmosphere (CH_4_,_pre_). Timestepping this model forward to the year 2014 and subtracting CH_4,pre_ reveals the additional CH_4_ component that has accumulated since 1700, due to equilibration with anthropogenically enhanced atmospheric pCH_4_ (CH_4,anthro_). Finally, subtracting the 2014 [CH_4_] distribution in this model from the full prediction with *J_prod_* enabled isolates the third component—CH_4_ that was produced biologically in the surface ocean by oxic CH_4_ production (CH_4_,_bio_).

### Future Oxic CH_4_ Production Prediction.

To assess how marine oxic CH_4_ production will respond to future depletion of surface ocean [PO_4_], we integrated our model forward from 2005 to 2300 while perturbing the model PO_4_ distribution. We adjusted the observed [PO_4_] distribution (*P_obs_*) each model year by applying the changes predicted by the Community Climate System Model (CCSM) under the RCP8.5 future climate scenario (37). We test two different methods, which apply either the absolute (Eq. **12**) or relative (Eq. **13**) change predicted by CCSM:

[12] $$[{\mathrm{PO}}_{4}]=\max\left(0,P_{\mathrm{obs}}+\left(P_{\mathrm{CCSM}}-P_{CCSM,2005}\right)\right),$$

[13] $$[{\mathrm{PO}}_{4}]=P_{\mathrm{obs}}\left(\frac{P_{\mathrm{CCSM}}}{P_{CCSM,2005}}\right).$$

Here, *P_CESM,2005_* and *P_CESM_* represent the CCSM PO_4_ distributions in the 2005 baseline year, and all succeeding years between 2005 and 2300, respectively. The perturbed [PO_4_] distribution is then incorporated into the parameterization of *J_prod_* (Eq. **10**), resulting in a prediction for oxic CH_4_ production in each future year (Fig. 5 and *SI Appendix*, Fig. S8).

## Supplementary Material

Appendix 01 (PDF)

## Data Availability

Model output discussed in manuscript is available for download from the Figshare archive at DOI: 10.6084/m9.figshare.29396495 (69). Model code is available from the Zenodo archive at DOI: 10.5281/zenodo.15875431 (70).
