# Supplementary material for: Phosphate scarcity governs methane production in the global open ocean
Source: Proc Natl Acad Sci U S A. 2026 Mar 17;123(12):e2521235123. doi: 10.1073/pnas.2521235123 (PMC13012047; doi:10.1073/pnas.2521235123)
Supplement: Supplementary file 1 — Appendix 01 (PDF) [file pnas.2521235123.sapp.pdf]

Supporting Information for
Phosphate scarcity governs methane production in the global open ocean

Shengyu Wang[1], Hairong Xu[1], Thomas Weber[1*]

[1]Department of Earth and Environmental Science, University of Rochester, Rochester, NY 14627, USA

Corresponding Author: Thomas Weber
Email: t.weber@rochester.edu

This PDF file includes:

Supporting text
Figures S1 to S9
Tables S1 to S2
SI References

Supporting Information Text

Supplementary Methods

Derivation for substrate-limited organic matter cycling parameterization: Our formulation for CH_4 production during microbial organic matter cycling (Eq. 9, main text) is derived by assuming that the decomposition of an organic substrate is limited by the substrate concentration (S) itself and the biomass of the microbial population (B_m). We begin by considering a generic population model for B_m:

$$\frac{dB_m}{dt} = \mu_{max}\frac{S}{S+K_S}B_m - m_1 B_m - m_2 {B_m}^2 \quad \text{(S1)}$$

Here, K_S is the substrate half-saturation constant, μ_{max} is the maximum microbial growth rate, and m_1 and m_2 are linear and quadratic mortality terms respectively. Assuming that CH_4 is produced during the conversion of substrate to biomass, then for a constant microbial growth efficiency and a constant CH_4 yield per unit of substrate consumption, the CH_4 production rate (J_{prod}) will be proportional to microbial growth i.e. the first term on the right-hand side of Eq. S1:

$$J_{prod} \propto \mu_{max}\frac{S}{S+K_S}B_m \quad \text{(S2)}$$

To develop a simple parameterization for J_{prod} without explicitly modeling the microbial community, we assume the population remains at quasi-steady state with the substrate concentration and solve Eq. S1 for B_m:

$$B_m = \frac{\mu_{max}}{m_2}\frac{S}{S+K_S} - \frac{m_1}{m_2} \quad \text{(S3)}$$

Finally, substituting Eq. S3 into Eq. S2 and grouping constants yields:

$$J_{prod} = a_s \frac{S}{S+K_S}\left(\frac{S}{S+K_S} - b_s\right) \quad \text{(S4)}$$

which is identical to Eq. 9 in the main text.

Correction of World Ocean Atlas PO_4: A recent compilation of high-precision phosphate measurements (1) illustrated that the World Ocean Atlas (WOA) [PO_4] climatology tends to be biased high at very low concentrations due to the limitations of traditional measurement methods, and the strength of this bias differs between oligotrophic regions. Because one of our model configurations attempts to resolve the relationship between CH_4 production and low [PO_4], we attempted to correct the bias in the WOA 2018 climatology ([PO_4]$_{WOA}$) to provide a more realistic [PO_4] input for the model. To achieve this, we determine a spatially-variable linear calibration relationship between [PO_4]$_{WOA}$ and high-precision measurements ([PO_4]$_{HP}$; (1)):

$$[PO_4]_{HP} = A_P[PO_4]_{WOA} + b_P \quad \text{(S5)}$$

First, we binned the [PO_4]$_{HP}$ data onto the WOA18 grid, and then separated data points between the Atlantic, Pacific, and Indian Oceans according to the WOA18 basin mask. The Pacific was further split into the North and South Pacific along the equator (there was insufficient data in the South Atlantic to facilitate a similar separation of the Atlantic). The calibration coefficients (A_P and b_P in Eq. S5) were then obtained for each region by linear correlation of coincident datapoints between the two datasets, and are provided in Table S2. Finally, the calibration relationships for each region were applied to [PO_4]$_{WOA}$ for all points with concentrations lower than 0.5 μM to yield a corrected global climatology, and the corrected [PO_4] distribution was then interpolated to our

model grid for use in Eq. 10 (main text). The corrected and corrected surface [PO_4] are compared in Fig. S9.

In a model sensitivity test, we repeated our optimization using the uncorrected PO_4 distribution in the parameterization for J_{prod} (Eq. 10), and found that the model was able to match the surface [CH_4] distribution just as well as the version with corrected PO_4 (Fig. S4b) – it simply selected a slightly different relationship between J_{prod} and [PO_4] (Table S1). This reveals that the PO_4 correction is not responsible for the PO_4-based parameterization outperforming other model configurations, but is preferable for accurately constraining the impact of very low [PO_4] on CH_4 production.

Hybrid CH_4 production parameterizations: While our study focused on testing each hypothesized environmental control on oxic CH_4 production in isolation, we also tested two "hybrid" parameterizations that each combined two different factors. First, we tested a parameterization for CH_4 production during MPn degradation that combines (by multiplication) the substrate limitation effect (Eq. 9, main text) with the PO_4 inhibition effect (Eq. 10, main text), yielding:

$$J_{prod} = a_{MPn} \left(.5 \times \tanh\left(\frac{([PO_4]-P_{crit})}{w}\right) + .5\right) \frac{S}{S+K_S}\left(\frac{S}{S+K_S} - b_s\right) \quad \text{(S6)}$$

Here, a_{MPn} represents a theoretical maximum production rate when PO_4 is scarce and the substrate (again represented by the semi-labile DOC pool) is replete, and all other parameters are as in Eq. 9 and Eq. 10 of the main text. This J_{prod} parameterization contains more free parameters than the others, and does not improve the model fit to observations relative to Eq. 10, suggesting that PO_4 inhibition is the critical factor controlling CH_4 production during MPn decomposition. Second, we tested a formulation that combines (by addition) the PO_4-inhibited MPn degradation pathway (Eq. 10, main text) and the photosynthetic pathway (proportional to NPP) to assess whether photosynthesis might contribute a weak "background" CH_4 production rate in addition to MPn cycling. This yields:

$$J_{prod} = a_{MPn} \left(.5 \times \tanh\left(\frac{([PO_4]-P_{crit})}{w}\right) + .5\right) + a_{NPP} * NPP \quad \text{(S7)}$$

In this formulation, the coefficient a_{NPP} optimized to zero, to prevent CH_4 supersaturation at high latitudes, again identifying [PO_4] as the primary control on CH_4 production.

Figures

Fig. S1.

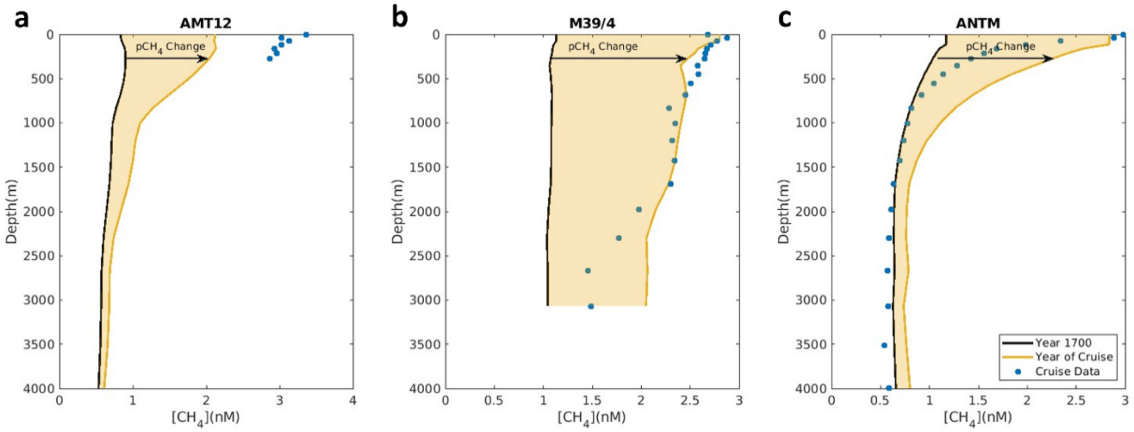

Fig. S1. Modeled vertical [CH_4] profiles from model configuration that excludes biological methane production for cruise transects: (a) AMT12 (mid-Atlantic), (b) M39/4 (North Atlantic), and (c) ANTM (Southern Ocean). The model broadly captures the observed [CH_4] distributions in high-latitude regions such as M39/4 and ANTM. However, it fails to reproduce the supersaturation observed in low-latitude regions like AMT12, highlighting the role of in situ methane production in these areas.

Fig. S2.

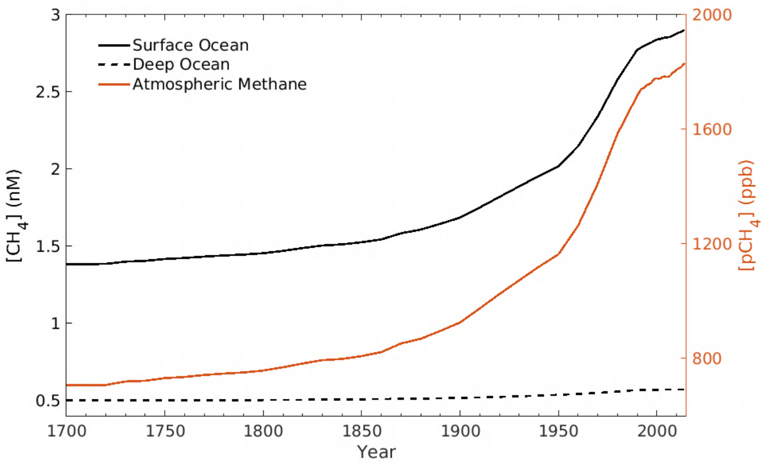

Fig. S2. Modeled global-mean oceanic CH_4 concentrations from 1700 to 2014, shown for the surface ocean (0–100 m, solid line) and deep ocean (>1000 m, dashed line). The orange line represents historical atmospheric pCH_4 levels (ppb) from Meinshausen 2017 (2). Over the past three centuries, atmospheric pCH_4 increased from 706 ppb to 1832 ppb. Driven by rapid air-sea exchange, the average surface ocean [CH_4] more than doubled from 1.03 nM to 2.55 nM. In contrast, the deep ocean shows only a modest increase, rising from 0.57 nM to 0.64 nM (a 12% change), reflecting the slow turnover and large volume of the deep ocean reservoir.

Fig. S3.

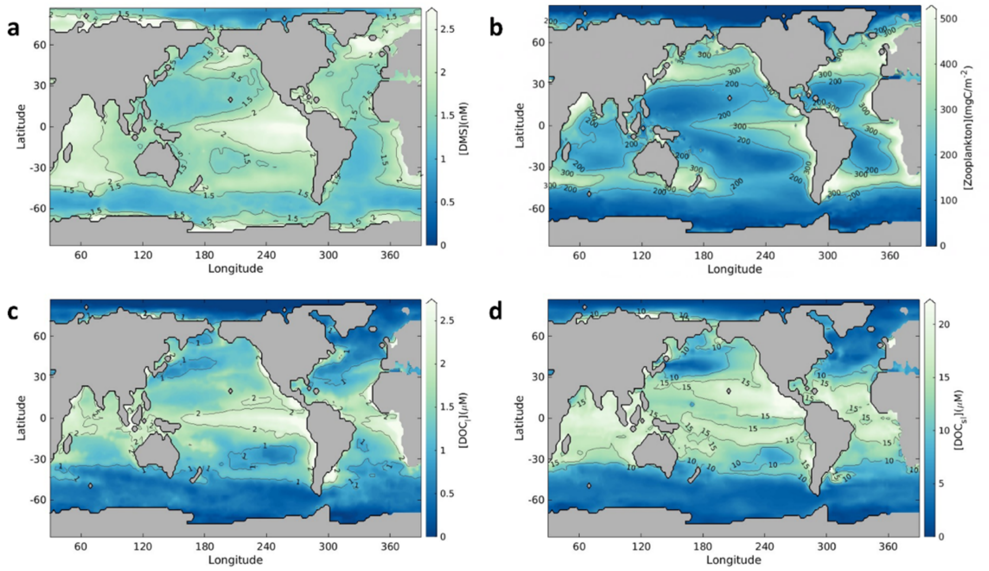

Fig. S3. Global surface distributions of properties that used to parameterize methane production (see Methods): (a) Dimethylsufide (DMS)(3), (b) zooplankton biomass from statistical extrapolation of data(4) (see methods), (c) semi-labile DOC(5), and (d) labile DOC(5).

Fig. S4.

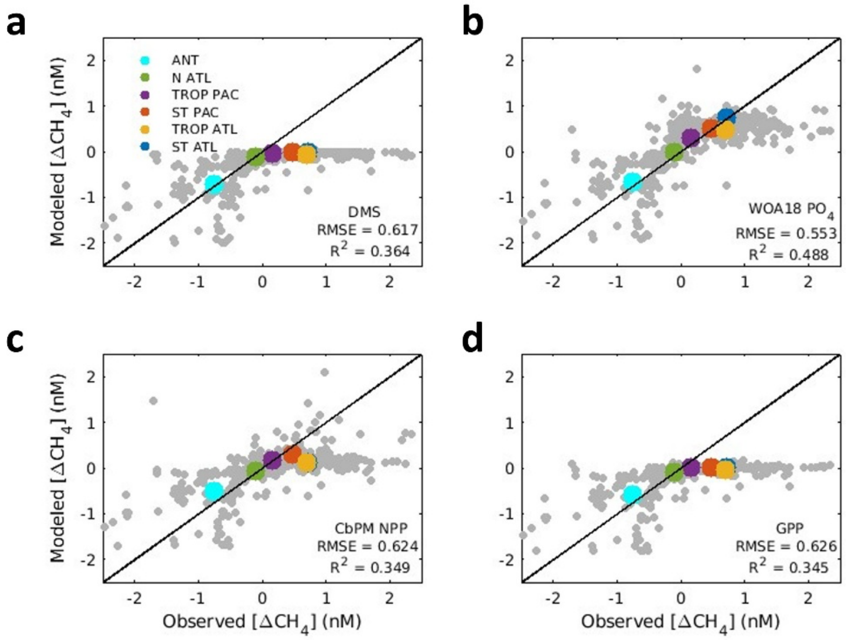

Fig. S4. Observed versus modeled CH_4 supersaturation (as in Fig. 2) for models using additional methane production parameterizations not illustrated in the main text: (a) CH_4 production during organic matter cycling (Eq. 9) using DMS as the organic substrate (see Methods), (b) phosphate-inhibited CH_4 production (Eq. 10) using uncorrected WOA18 phosphate data, (c) Photosynthetic CH_4 production (Eq. 7) using CbPM-derived Net Primary Production (NPP)(6), and (d) Photosynthetic CH_4 production (Eq. 7) using Gross Primary Production (GPP) instead of NPP parameterization (7).

Fig. S5.

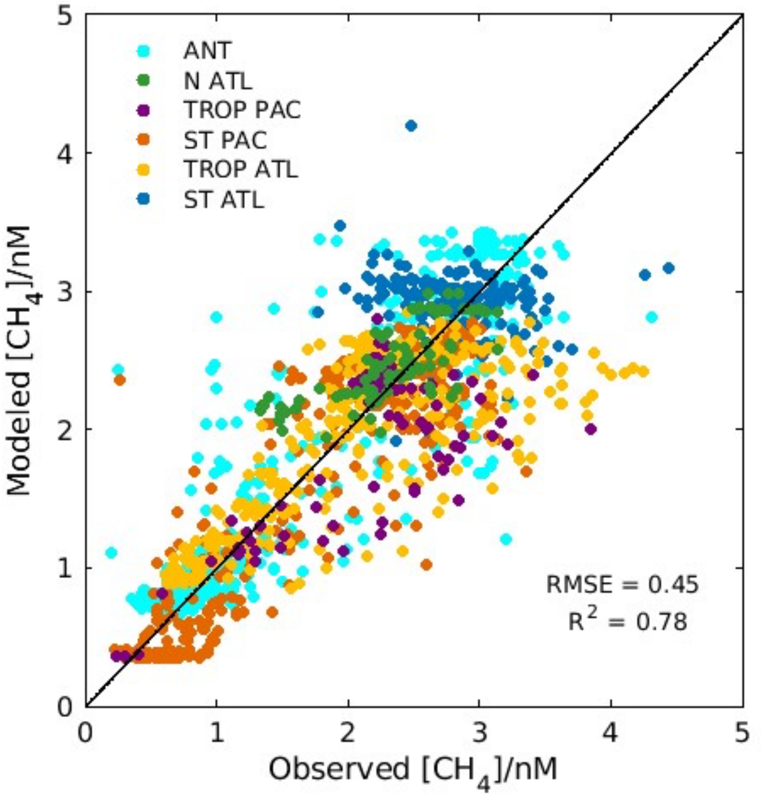

Fig. S5. Global model-data comparison for [CH_4] across all depths, from model using PO_4-based parameterization for methane production. Clustering of points around the black 1:1 line, with no systematic biases, demonstrates that this model configuration is broadly consistent with observations across all regions and depths.

Fig. S6.

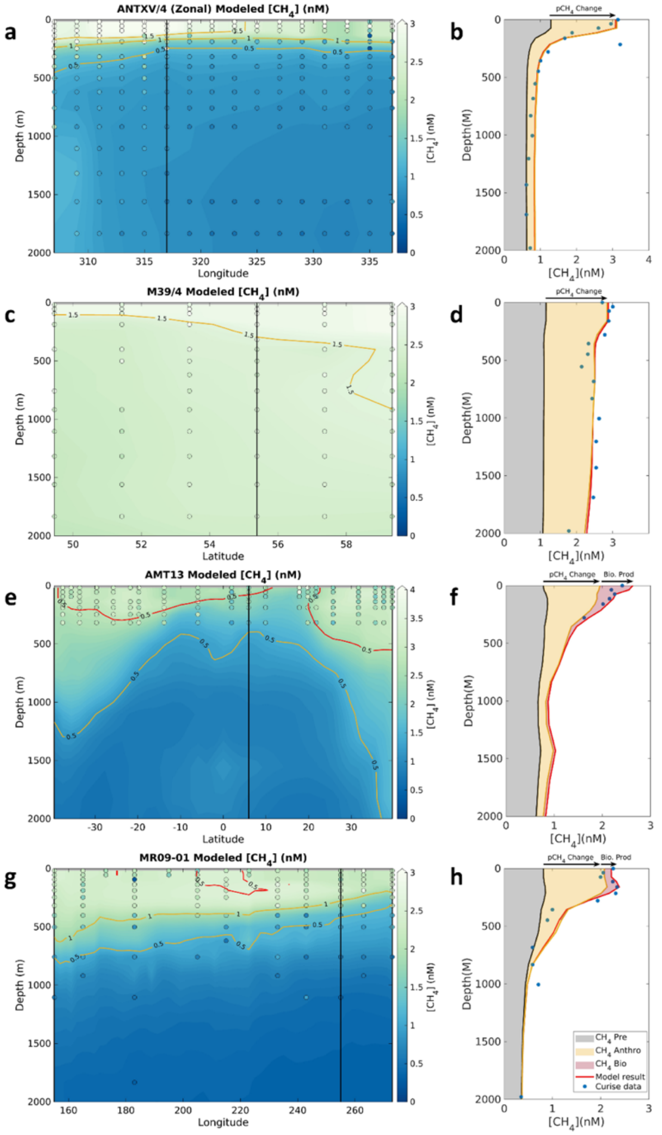

Fig. S6. Comparison between cruise observations and model simulated [CH_4], extending the analysis presented in Fig. 3 of the main text to three additional cruise sections. This figure includes: (a-b) a zonal section from cruise ANTXV/4 (ANTZ) in the Southern Ocean, (c-d) meridional cruise M39/4, south of Greenland in the North Atlantic, (e-f) meridional cruise AMT13 in the low-mid latidue Atlantic, and (g-h) zonal cruise MR-09-01 in the Tropical Pacific. All cruise tracks are shown on Fig. 1a. Red contours indicate biologically-produced methane ($CH_{4,bio}$), while orange contours represent the enhancement due to due to invasion of anthropogenic CH_4 ($CH_{4,anthro}$), both plotted by 0.5 nM increment intervals (as in Fig. 3). The model successfully captures the observed CH_4 distributions and sheds light on the origins of the observed CH_4. In high-latitude regions (cruises ANTZ and M39/4), biological production is minimal, and methane vertical CH_4 are dominated by invasion of anthropogenic CH_4. In contrast, low latitude regions (cruises AMT13 and MR-09-01) exhibit accumulation of biologically-produced CH_4 in the surface, which is most apparent in PO_4-starved Atlantic (AMT13) and less pronounced in the relatively PO_4-replete Tropical Pacific (MR-09-01).

Fig. S7.

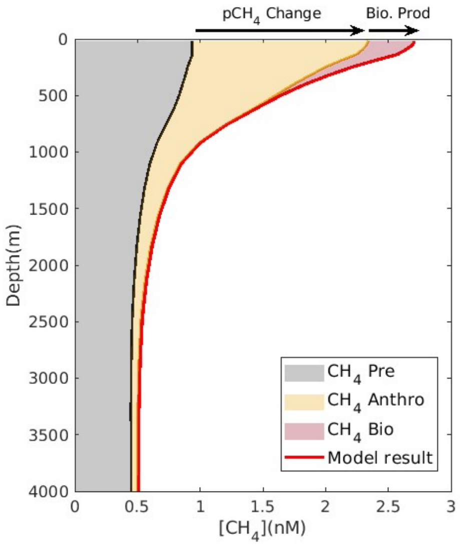

Fig. S7. Global-mean vertical profiles of modeled CH_4 concentration, broken into preindustrial, anthropogenic, and biologically produced components. While individual cruise sections are shown in Fig. 3 and Fig. S6, this global-mean profile provides context for the large-scale contributions of each component, as referenced in the main text.

Fig. S8.

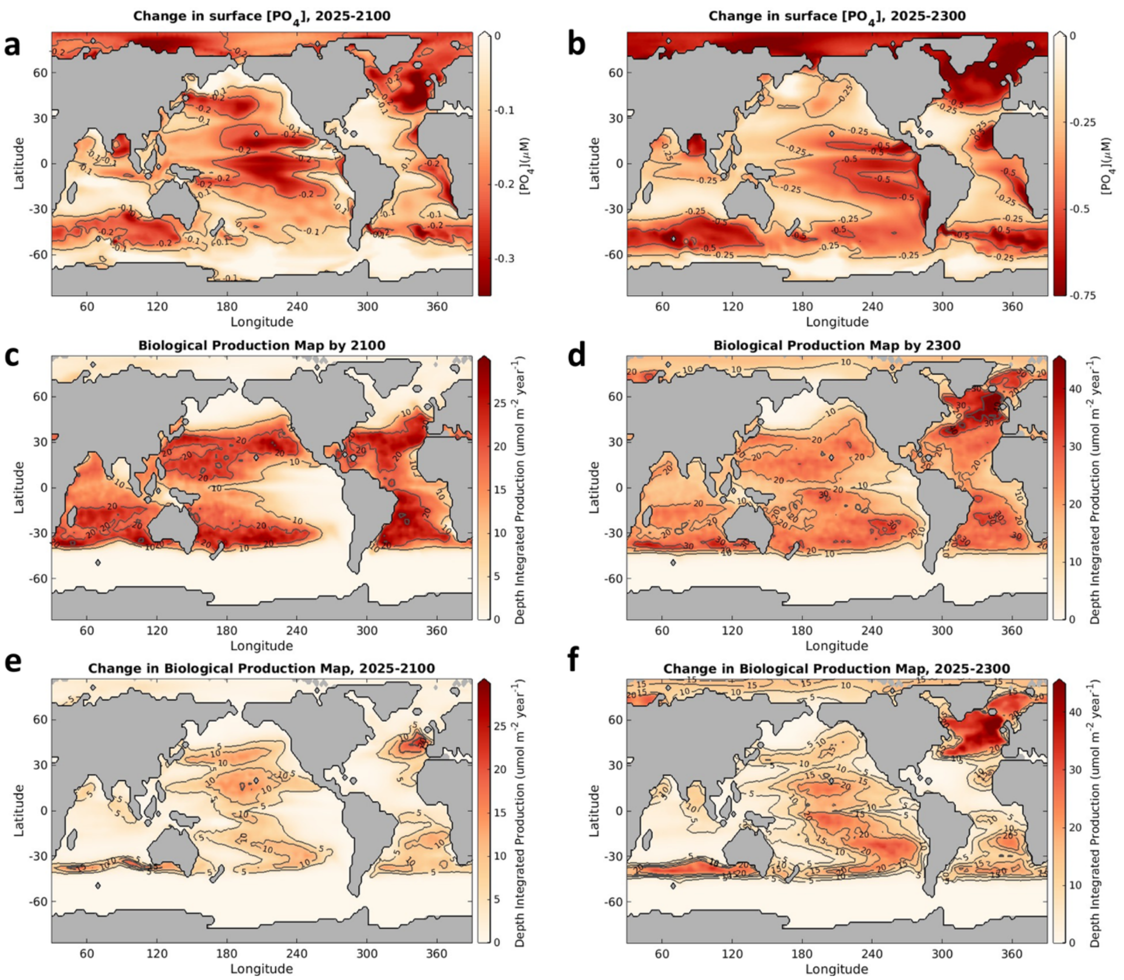

Fig. S8. Projected future changes in surface PO_4 and biological CH_4 production in the open ocean. (a-b) Changes in surface PO_4 concentrations (0–100 m average) relative to 2025, projected for the years 2100 (a) and 2300 (b)(8). (c-d) Biological methane production rates projected for the years 2100 (c) and 2300 (d), by applying our optimized parameterization to the perturbed PO_4 distribution (see Methods). (e-f) Change in biological methane production fluxes between 2025 and 2100 (e), and between 2025 and 2300 (f).

Fig. S9.

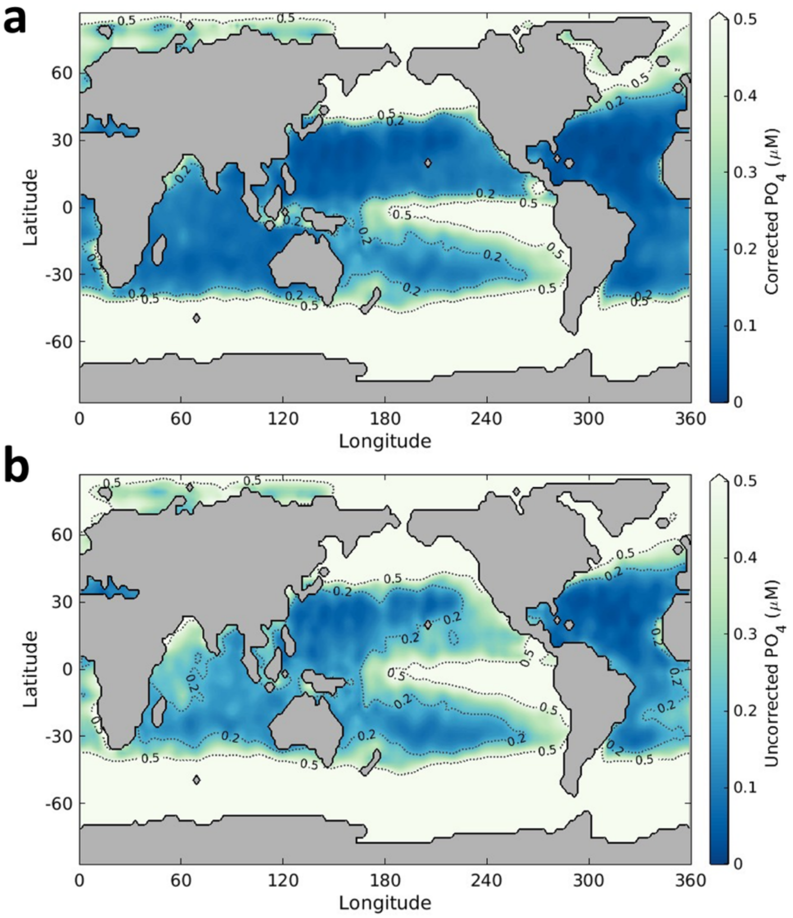

Fig. S9. Global surface distribution of phosphate concentrations: (a) corrected phosphate data based on Martiny et al.(1), and (b) annual mean phosphate concentrations from the World Ocean Atlas 2018 (WOA18)(9).

Tables

Table S1. Parameters of the global CH4 cycle model, including initial selections and optimized values following data assimilation.

Model configuration	Parameter	Meaning	Unit	Initial Value	Optimized Value
All	k_{ox}	aerobic oxidation rate constant	yr^{-1}	1/100	1/56—1/338[a] (1/172)[b]
	C_{crit}	threshold concentration beneath which oxidation ceases	μM	0.5	0.183—0.522[a] (0.324)[b]
NPP-based production	a_m	CH_4 yield constant from microplankton	μmol CH_4 gC^{-1} m^{-1}	0.1	0.0001
	a_n	CH_4 yield constant from nanoplankton	μmol CH_4 gC^{-1} m^{-1}	0.1	0.0001
	a_p	CH_4 yield constant from picoplankton	μmol CH_4 gC^{-1} m^{-1}	0.1	0.324
Zooplankton	a_{zoo}	CH_4 production rate per unit biomass	μmol CH_4 gC^{-1} m^{-1}	0.1	0.07
	Q_{10}	temperature-sensitivity constants	unitless	2	4
	kO_2	oxygen half-saturation constant	μM	0.5	0.085
DOC Labile	a_s	maximum production rate	μmol CH_4 m^{-3} yr^{-1}	50	56.445
	b_s	threshold effect (see eq.9)	μM	0.5	0.758
	K_s	Half-saturation concentration	μM	0.5	0.363
DOC Semi-Labile	a_s	maximum production rate	μmol CH_4 m^{-3} yr^{-1}	50	45.941
	b_s	threshold effect (see eq.9)	μM	0.5	5.421
	K_s	Half-saturation concentration	μM	0.5	0.348
DMS	a_s	maximum production rate	μmol CH_4 m^{-3} yr^{-1}	50	16.341
	b_s	threshold effect (see eq.9)	μM	0.5	98.728
	K_s	Half-saturation concentration	μM	0.5	0.862
PO_4-based production	a_0	maximum methanogenesis rate when PO_4 is depleted	μmol CH_4 m^{-3} yr^{-1}	30	21.809 (20.936)[c]

	P_{crit}	critical $[PO_4]$ concentration at the center of the hyperbolic tangent function	μM	0.5	0.206 (0.275)[c]
	w	how sharply production declines as a function of [PO4]	unitless	3	4.965 (3.485)[c]

[a]Range of values selected by different model configurations
[b]Value selected by most skillful model configuration (with PO_4-inhibited CH_4 production)
[c]Value selected for model using uncorrected rather than corrected $[PO_4]$

Table S2. Region-specific calibration parameters for correcting WOA [PO4], based on correlation to high precision measurements (Eq. S5).

Region	A	b	# of data points
North Pacific	0.882	-0.030	903
South Pacific	1.032	-0.038	982
Indian Ocean	0.773	-0.030	79
Atlantic	0.693	-0.018	2638

SI References

1. A. C. Martiny *et al.*, Biogeochemical controls of surface ocean phosphate. *Sci Adv* **5**, eaax0341 (2019).
2. M. Meinshausen *et al.*, Historical greenhouse gas concentrations for climate modelling (CMIP6). *Geosci Model Dev* **10**, 2057-2116 (2017).
3. W. L. Wang *et al.*, Global ocean dimethyl sulfide climatology estimated from observations and an artificial neural network. *Biogeosciences* **17**, 5335-5354 (2020).
4. T. D. O'Brien, COPEPOD: The Global Plankton Database. An overview of the 2014 database contents, processing methods, and access interface. U.S. Dep. Commerce, NOAA Tech. Memo., NMFS-F/ST-37, 29 pp.
5. M. Nowicki, T. DeVries, D. A. Siegel, Quantifying the Carbon Export and Sequestration Pathways of the Ocean's Biological Carbon Pump. *Global Biogeochem Cy* **36** (2022).
6. M. J. Behrenfeld, E. Boss, D. A. Siegel, D. M. Shea, Carbon-based ocean productivity and phytoplankton physiology from space. *Global Biogeochem Cy* **19** (2005).
7. Y. Huang, D. Nicholson, B. Huang, N. Cassar, Global Estimates of Marine Gross Primary Production Based on Machine Learning Upscaling of Field Observations. *Global Biogeochem Cy* **35**, e2020GB006718 (2021).
8. J. K. Moore *et al.*, Sustained climate warming drives declining marine biological productivity. *Science* **359**, 1139-1142 (2018).
9. H. E. Garcia *et al.*, *World Ocean Atlas 2018, Volume 4: Dissolved Inorganic Nutrients (phosphate, nitrate and nitrate+nitrite, silicate)*, NOAA Atlas NESDIS 84 (NOAA National Centers for Environmental Information (NCEI), Silver Spring, MD, 2019), pp. 35.
